# Design of a Pharmacy Curriculum on Patient Centered Communication Skills

**DOI:** 10.3390/pharmacy9010022

**Published:** 2021-01-15

**Authors:** Majanne Wolters, Jacqueline G. van Paassen, Lenneke Minjon, Mirjam Hempenius, Marie-Rose Blokzijl, Lyda Blom

**Affiliations:** Division Pharmacoepidemiology & Clinical Pharmacology, Faculty of Science, Utrecht University, P.O. Box 80 082, 3508 TB Utrecht, The Netherlands; j.g.vanpaassen@uu.nl (J.G.v.P.); l.minjon@uu.nl (L.M.); m.hempenius@uu.nl (M.H.); m.r.m.blokzijl@uu.nl (M.-R.B.); a.t.g.blom@uu.nl (L.B.)

**Keywords:** patient cent(e)red communication, pharmacy, curriculum design, communication skills, teaching methods, assessment, pharmaceutical consultation, clinical medication review, patient centered care

## Abstract

For delivering high quality pharmaceutical care pharmacy students need to develop the competences for patient centered communication. The aim of the article is to describe how a curriculum on patient centered communication can be designed for a pharmacy program. General educational principles for curriculum design are based on the theories of constructive alignment, self-directed learning and the self-determination theory. Other principles are paying systematic and explicit attention to skills development, learning skills in the context of the pharmacy practice and using a well-balanced system for the assessment of students’ performance. Effective educational methods for teaching communication skills are small group training sessions preferably with (simulation) patients, preceded by lectures or e-learning modules. For (formative or summative) assessment different methods can be used. The Objective Structured Clinical Exam (OSCE) is preferred for summative assessment of communication competence. The principles and educational methods are illustrated with examples from the curriculum of the master Pharmacy program of Utrecht University (The Netherlands). The topics ‘pharmaceutical consultations on prescription medicine,’ ‘pharmaceutical consultations on self-care medication’ and ‘clinical medication reviews’ are described in detail. Finally, lessons learned are shared.

## 1. Introduction

In many countries, the primary focus of the pharmacist shifted from ‘product’ to ‘patients’ in the last twenty to thirty years [1]. This paradigm shift is captured in the concept of pharmaceutical care which urges pharmacists to take responsibility for the clinical outcomes of drug therapy by preventing, identifying and resolving drug related problems [2]. In the Strategic Plan of the International Pharmaceutical Federation (FIP) of 2019 this shift is supported by the following strategic outcome: ‘*support and empower pharmacists to provide high quality person-centered pharmaceutical care to improve health outcomes for individuals and populations alike*’ [3]. To deliver this high quality pharmaceutical care patient centered communication is necessary [4,5]. To prepare students for this task pharmacy curricula should pay attention to communication with patients [6,7].

As authors we are working (LB has worked) at the Department of Pharmaceutical Sciences at Utrecht University, the Netherlands. In 2016, a new curriculum for the master of Pharmacy was implemented at our institution to fit with current developments of the pharmaceutical profession in the Netherlands. The general learning outcomes of our master curriculum are based on the CanMEDS framework of which communication is one of the domains [8].

In this article, we describe how a curriculum on patient centered communication (as part of a pharmacy curriculum) can be designed based on general educational principles and effective educational methods aimed at preparing students for their role as pharmaceutical care providers. Examples from our own master curriculum illustrate how we have translated these principles into practice. These examples are meant as a source of inspiration without claiming to be the gold standard.

In Section 2 we briefly describe how we have defined the concept of patient centered communication. Section 3 provides an outline of general educational principles. The specific methods for teaching and assessing communication skills are elaborated on in Section 4. The three topics of our master program on patient centered communication are described in more detail in Section 5.1 pharmaceutical consultations on prescription medicine, Section 5.2 pharmaceutical consultations on self-care medication and Section 5.3 clinical medication reviews. Finally, some general remarks are made.

## 2. Patient Centered Communication

**Figure 1:**
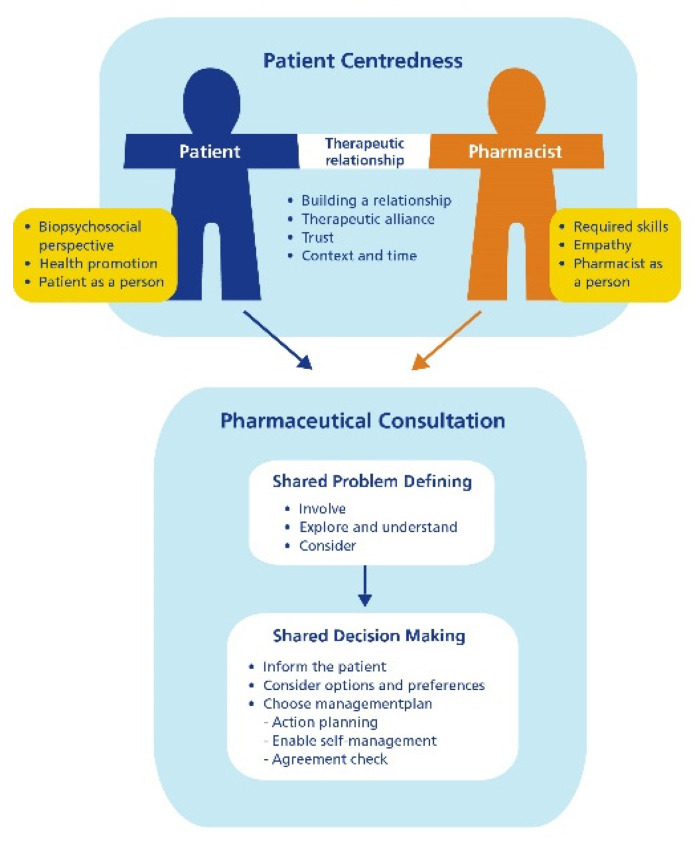
Utrecht Model for Patient centered communication in the Apotheek (UMPA) model: Utrecht Model on Patient centered communication in the pharmacy about drug related problems.

Patient centered communication is defined as ‘providing care that is respectful of and responsive to individual patient preferences, needs and values and ensuring that patient values guide all clinical decisions’ [9]. It is seen as a useful method for effective care [10,11]. Our institution developed, based on a scoping review, a model for patient centered communication in the pharmacy practice, the UMPA model (Utrecht Model for Patient centered communication in the Apotheek (-/= Dutch for Pharmacy) (Figure 1) [12].

According to this model, the three main elements of a patient centered consultation are (i) building a therapeutic relationship with the patient, (ii) defining the problem together with the patient and (iii) coming to a shared decision on the pharmaceutical care plan.

Based on the scoping review, we defined key points of a patient centered approach (Box 1) [12].

Box 1 **Key points of a patient centered approach as used in teaching.**

- The pharmacist considers the patient as a unique person, with unique problems which require tailored solutions
- The focus of the pharmacist is more on the patient than the product, the pharmacist is respectful and empathic
- The pharmacist strives for mutual understanding and agreement, both pharmacist and patient participate: two-way communication
- The pharmacist shares control and responsibility with the patient as much as possible, respecting the patient’s right on information
- The pharmacist stimulates the patient to be self-reliant
- The pharmacist adapts their communication style to the individual patient
- The pharmacist views being competent in communication as an important part of their profession

The communication skills future pharmacists need to learn for practicing such a patient centered approach are listening, building rapport, non-verbal communication, questioning (open questions if possible), paraphrasing, summarizing, reflecting, presenting information, giving advice, confronting and structuring the conversation [13,14,15].

Models or frameworks for communication skills help structure the skills that are being taught [16,17]. This is relevant for both the teaching staff as the students. As general models for patient centered communication we use the UMPA-model and the (concept) professional guideline of the KNMP (The Royal Dutch Pharmacists Association), which is based on the widely accepted Calgary Cambridge model [12,18,19].

## 3. General Educational Principles for Our Curriculum Design

To design a coherent curriculum that is grounded in educational research, it is important to use a set of guiding principles. At our institution, the following six guiding principles are used [20]. Next to a description, examples are given to illustrate how these principles were translated to our curriculum on patient centered communication. These examples will be discussed in depth in Section 4 and Section 5.

- *Systematic and explicit attention to the development of academic and personal skills and values*. Patient centered communication is repeatedly trained and practiced in courses in year 1 and 3 of our master program, increasing the complexity of the specific skills or consultations over time.
- The principle of *constructive alignment*: a curriculum in which the teaching activities are based on the desired learning outcomes, which are assessed in such a way that the desired study behavior of students will be evoked [21]. Consequently, it is important to describe the learning objectives carefully. As a source of inspiration, we used Bachmann’s description of learning objectives for a core communication curriculum for health care professions [22]. This description is based on literature and expert knowledge.
- *Learning in context:* knowledge and skills are taught in a context relevant for the future profession. Therefore, it is important to integrate communication competence with pharmaceutical expertise in educational activities and not provide it as a single course only focusing on communication skills. By incorporating communication skills training in different courses, a wide variety of contexts is offered. Some of our courses use the method of experiential learning. This means that learning activities at the university run parallel with students’ internships at the pharmacy. It aims to enhance the integration of theoretical understanding and practical experience and to promote transfer of learning. During internships students can practice what they have learned and get feedback from their internship supervisors, which is an effective way of learning [13].
- The *Self-Determination Theory (SDT)* poses that people will be motivated when three psychological needs are fulfilled, namely feeling competent, feeling connected with others and being able to make autonomous decisions [23]. Therefore, the program should stimulate active study behavior, it should be challenging and varied and should enable students to follow individual interests, thus motivating students for learning.
  The courses in our curriculum in which communication skills training is integrated are all compulsory, so students do not have the freedom to opt out. Nevertheless, we stimulate, activate and motivate students to participate as active learners in different ways. Firstly, we offer students e-learning modules that they can use to prepare for the training session. This gives them the freedom to choose when to do these modules. Secondly, some learning activities (regarding clinical medication reviews) are optional. Thirdly, students can choose their own learning goals for the training sessions. Finally, students have multiple opportunities to pass the assessment. In time, they will be able to succeed and feel competent.
- *Using a well-balanced system of mentoring and assessment.* We try to create such a balanced system by using different types of assessment (self-reflection assignments, peer feedback, assessment of consultations), as well as both formative (focused on development) as summative assessment (focused on meeting the defined standards). We also pay attention to reflection through a system of mentoring by qualified teachers.
- *Self-directed learning:* the direction of the learning process is gradually shifted from teacher to student, from more traditional forms of teaching to methods that stimulate students to take responsibility for their own learning process [24]. At the beginning of year 1, we strongly guide our students in the educational activities followed by a summative assessment. Later in year 1 and 3 students have more responsibility for their own learning process. Then, the assessment is more often formative and the portfolio with personal reflections is even more important for their learning process.

## 4. Teaching Methods for Communication Skills

This paragraph describes different methods that can be used to design a coherent curriculum for communication skills training and assessment and which ones of these we used. Studies on how communication skills training and assessment are integrated in pharmacy curricula recommend to (i) use an evidence-based framework, (ii) start early followed by reinforcement over years, (iii) integrate communication training in the program with other learning activities, (iv) use appropriate assessment and (v) provide experiential training (with real or simulation patients) in the context of the pharmacy practice [25,26,27,28].

We used Miller’s Pyramid to order the different learning objectives and matching educational methods in our own curriculum [29]. Miller’s Pyramid consists of four stages: knows what and why, knows how, shows how, does (Table 1). This framework is used to design teaching programs [30].

To determine whether students can communicate in a patient centered way, we should not only assess their theoretical knowledge about effective communication but also assess how they communicate in practice. Similarly, teaching should not only focus on acquiring knowledge but also on learning the necessary skills and acquiring a professional attitude [30,31]. Thus, practical training and feedback, as well as reflective thinking and social interaction should be part of the learning process [13].

Table 1 describes how patient centered communication can be taught and assessed in the different stages of Miller’s Pyramid and which methods we use in our curriculum. Lectures and written materials are suitable for knowledge transfer but are not effective in developing communication skills [28,32,33]. Previous research has pointed out that communication skills training is an effective teaching method [33,34,35,36,37]. Skills training can have an effect on students’ confidence, knowledge, skills and attitudes [37]. Effective communication skills training should ideally contain instruction and demonstration, practice, (video) feedback and reflection [32,36]. In several pharmacy curricula, small group training sessions with simulation patients are offered [38,39,40,41].

In recent years, e-learning methods have also been incorporated in teaching programs for healthcare professionals. These have proven to be effective, especially as blended learning (combination of face-to-face and online learning activities) [42,43].

Finally, internships can be a valuable learning space for patient centered communication, offering the students the opportunity to repeatedly communicate with real patients in diverse situations.

In the following sections we will take a closer look at the preparatory e-learning modules, small group training and assessment and at how these are applied in our curriculum in relation to what is known from research.

### 4.1. Preparatory E-Modules

Since e-learning can be effective in combination with face-to-face learning, we integrated e-modules in the courses. In our former curriculum, we experienced that the preparation for training sessions that we offered did not suffice. Not all students read the written materials but more importantly the lectures and written material did not provide the student with knowledge and insight in the relevance of and the approach to patient centered communication. Therefore, they did not have a clear image of what an effective consultation would look like and their personal learning goals were often vague or general. Moreover, in general there can be a gap between the knowing and showing stages of Miller’s Pyramid: the required step is too big and frightening. To bridge this gap, we provide students an opportunity for self-study through the e-modules (Box 2). (Access to a demo module can be requested from the corresponding author MW.) The students highly appreciate these modules and, in our experience, serve the purpose [44].

Box 2 **Content of preparatory e-modules.**

- Theory: texts and knowledge clips
- Videoclips of (effective and not effective) consultations with reflection assignments
- Assignments to self-test both pharmaceutical knowledge as well as insight in consultations and communication
- Interactive simulation with a virtual patient
- Assignments to formulate personal learning goals and questions

### 4.2. Small Group Training (including Roleplay)

As stated earlier, communication skills training in small groups is an effective method to develop communication skills. However, working with small groups is expensive. So, for the optimal duration and group size of training sessions, a balance between effectiveness and efficiency has to be determined. A meta-analysis showed that the total number of attendees was significantly negatively correlated with the effect of the communication skills training, which supports the importance of small groups [37].

We offer our students small group training sessions of two to four hours. In most training sessions students practice with a simulation patient (group size 6–8 students) but sometimes they practice with one another (group size 12–15 students). They receive feedback from fellow students, simulation patients and teachers. In our experience, students not only learn from practicing themselves but also from observing each other and giving feedback. The duration of the training sessions provides sufficient opportunities to deal with various situations and problems.

#### 4.2.1. Importance of a Safe Learning Environment

Taking part in a role play is a challenging experience for almost all students. Therefore, it is important that students feel free to practice and dare to make mistakes or to be insecure. In order to create a safe environment, teachers are empathic, especially when students have problems with handling difficult situations, are frustrated or experience resistance themselves. We also pay attention to the phrasing of the feedback. We try to avoid labelling students’ behavior as ‘good’ or ‘bad’ but connect the effect of their behavior during role play to the goal at that point in the consultation. Working with small training groups provides each student the opportunity to practice, without being watched by a large group. On the other hand, it also prevents students from being safely out of the way as an observer all the time.

#### 4.2.2. Working with Simulation Patients

Simulation patients are used in both education and assessment. There is a difference between acting for learning or for assessment. In learning, the patient tunes in to the individual student to create an appropriate learning experience (zone of proximal development). During an assessment, because of reliability and validity, the approach to a case must be standardized, adjusted to the learning objectives which are to be assessed.

Practicing with simulation patients can have a significant impact on students’ communication skills over time [45]. Students appreciate working with simulation patients, because they can portray a patient in a realistic way [45,46,47,48]. On the other hand, practicing with peers is also beneficial, as students become more empathic by taking the patient’s perspective [49].

### 4.3. Assessment of Communication Competence

To assess students’ competence in patient centered communication, different methods are used (Table 1). The most common types of assessments are written exams, written reflection assignments, peer-feedback, feedback from internship supervisors, assessment of videos of real life pharmaceutical consultations and the so called OSCE, Objective Structured Clinical Exam [27,28]. These assessments can be used both formatively and summatively. In our curriculum, we use video feedback, written reflections, peer-feedback, feedback from simulation patients and OSCEs as formative assessment methods. Of these, OSCEs and assessments of learning activities during internships (recorded in student portfolios) are used as summative assessment methods.

(Summative) assessment drives students learning [33]. Therefore, the assessment should fit the intended learning outcome. The following types of commonly used summative assessment have their strengths and weaknesses and each type can have a valuable function in a different phase of the learning process.

*A written exam* on relevant communication and behavior theory was found to be associated with students’ performance in an OSCE [50]. This could be the result of the students knowing (what and why) and knowing how to communicate effectively (stage 1 and 2 of Miller’s Pyramid). However, it is not advisable to use written exams as single summative assessment method, because of its limitations in predicting actual performance. Therefore and to limit the number of assessments, we do not include a written exam in our curriculum.

*Students’ self-assessment and written reflections* are used to assess communication skills in some curricula [1]. A disadvantage is that being able to reflect well on one’s own behavior is a separate skill from actual communication skills. Therefore, this type of assessment is less suitable as summative assessment [27] and serves better as a method for formative assessments, as students will benefit from critically reflecting on their own performance.

*Real-life consultations* with real patients (during internships) are interesting because it offers the possibility to assess actual performance (stage 4 of Miller’s Pyramid). However, these diverse consultations are difficult to compare and grade. Assessment of real-life consultations is useful in later stages of the curriculum, when students are more self-directed learners and assessment is not focused on strict pass or fail. In case the internship supervisors have an important role in the assessment, they need to be competent in both communication and assessment, which may require careful instruction and training [13].

The *OSCE* is a widespread method that is used to assess health professionals’ competence in patient centered communication [51,52,53]. An OSCE is a performance-based test in which students go through a series of stations in which they consult with standardized patients who present a specific problem. The students are observed and evaluated by teachers or experts who use standardized criteria for assessment. An OSCE is a suitable way to assess whether a student can show the required communication skills. Therefore, schools of pharmacy are interested to implement OSCEs in their curricula [28,54].

## 5. Major Topics on Patient Centered Communication in Our Curriculum

An important focus of our master program is the pharmacist as a health care provider. The educational activities concerning communication with patients involve three professional activities: pharmaceutical consultations on prescription medicines, pharmaceutical consultations on self-care medication and clinical medication reviews.

### 5.1. Pharmaceutical Consultations on Prescription Medicines

Performing pharmaceutical consultations on prescription medicines is a key task of Dutch community pharmacists. Therefore, a lot of attention is paid to this throughout three different courses in the first half year of the master program before students have their first internship in a community pharmacy. Six different topics that are relevant for the pharmacy practice are taught in these courses (Table 2).

In the first two courses, students learn how to deal with standard consultations in which medication is delivered for the first time (topic 1) or a repeat prescription (topic 2). Students learn the basic structure of these consultations, how to elicit patients’ situation and needs, how to provide information in an effective manner and how to reach a shared decision (as patient and pharmacist) on medication use, based on the patient’s situation and preferences (Table 3).

Students also learn how to deal with communicatively challenging situations (topic 3–6): dealing with a distracted, talkative or emotional patient or addressing non-compliance. The patient cases used for these training sessions are about chronic and psychiatric diseases, which are also the topics of these courses. After the teaching/learning activities on the six topics, students take part in a formative OSCE. This formative assessment gives the students the opportunity to experience what an OSCE is like and also become aware of their current ability. Students who perform well on a specific station at the formative OSCE get an exemption for that station in the summative assessment. This is more time and cost efficient and it motivates the students to prepare for the formative assessment.

In the third course, in which the summative OSCE is taken, students run multiple simulation pharmacies in groups of five to seven students as a preparation for their first internship [55]. This gives them the opportunity to practice their communication and pharmaceutical skills with simulation patients who visit these mock pharmacies. Students also get the opportunity to practice individually for half an hour with a simulation patient and receive extensive verbal feedback; they can choose what kind of station and/or skill they want to practice. So they have multiple opportunities to prepare for the summative OSCE.

Students who fail one or more stations in the summative OSCE retake the failed stations later on. Those who have not passed all the stations of the OSCE after the retake, take part in an individual remedial teaching program to prepare them for the specific stations they have to pass. This program consists of three meetings of 45 minutes and is focused on their personal learning goals.

### 5.2. Pharmaceutical Consultations on Self-Care Medication

Dispensing and advising on self-care medication is an important task for pharmacists, because it enables them to provide accessible care. Delivering self-care medication is covered in year 1 before students’ first internship in a community pharmacy (Table 4).

What students have learned for pharmaceutical consultations on prescription medicines gives a firm basis for delivering self-care medication, since the necessary communication skills and the structure of the consultation are the same (Table 3). The main difference with delivering prescribed medicines is that students have to determine whether self-care medication is appropriate in the patient’s situation. They need clinical reasoning skills to form a working diagnosis about a patient’s health problem in order to advice whether self-treatment is an option and what kind of drug treatment or non-drug approach is suitable. Students are taught to use the so-called WHAM-questions. This acronym stands for Who is it for?; How long have the symptoms been present, (what are the symptoms)?; What Actions have been taken so far?; What other Medication is being used at present? Using these questions helps pharmacists gather the information needed to give advice according to the self-care guidelines. (Note that in other countries the acronym WWHAM is used, which is slightly different but serves the same purpose) [56].

After a lecture and small group training, students practice self-care consultations with simulation patients in the previously mentioned simulation pharmacies. In addition, every student is also visited once by a simulation patient, with whom they practice a self-care consultation (30 min in total) and receive feedback based on their personal learning goals. This serves as a formative assessment of self-care consultations.

During their first internship students are formatively assessed by their internship supervisor on delivering self-care medication. This is one of the learning activities on which the students are assessed during their internships.

### 5.3. Clinical Medication Reviews

It is widely known that patients may experience problems when using medication or their treatment is suboptimal [57]. Therefore new pharmaceutical care services are being developed. The Clinical Medication Review (CMR) has been implemented and researched in many countries, countries use different descriptions [58,59,60,61,62,63]. According to the Dutch multidisciplinary guideline ‘Polypharmacy in the elderly’ and the guideline ‘Clinical Medication Review’ of the KNMP, Royal Dutch Pharmacist Association, pharmacists and general practitioners are obliged to perform CMRs in older patients, with the aim of optimizing the medication of patients [64,65] (Box 3).

Since CMRs are part of the Dutch pharmaceutical care services, more attention has been given to this topic in our curriculum since 2016. Students learn the whole process of a CMR, in the context of this publication we only focus on the consultations with the patient (Table 5).

In year 1, the students learn a conversation model for patient interviews (Table 6). The aim of this interview is to identify drug related problems (DRPs), that are relevant for the patient’s well-being. Students in year 1 are expected to identify possible DRPs, based on their analysis of the medical record and the information they gather during the interview. However, because of the complexity, we do not expect them (as novices) to be able to address these issues and give advice to the patients already during the interview. In year 3, we expect students to be able to not only identify DRPs but also to prioritize and decide which issues the students as pharmacists can already tackle themselves, without consulting the physician. This requires not only pharmacotherapeutic expertise, including clinical reasoning but also awareness of their professional role as a pharmacist.

In year 3 students learn how they can use the method of motivational interviewing (MI) when discussing the pharmaceutical care plan with the patient. MI is ‘a collaborative, person-centered form of guiding to elicit and strengthen motivation for change’ [66]. MI is included in our curriculum, because it can be helpful in discussing the pharmaceutical care plan with the patient, especially when patients are hesitant or ambivalent towards changing their medication. MI in general also provides tools to take on the role of a health coach for patients, providing patient centered care [67,68].

Through all these activities during their master, students have grown in expertise and professionalism. In their final internships their level of patient centered communication should be adequate for a starting pharmacist.

Box 3 **Definition and structure of a Clinical Medication Review (grey: consultations with a patient).**

A Clinical Medication Review (CMR) is defined as ‘a structured critical examination of a patient’s drug treatment’ in which ‘both pharmacist, general practitioner (GP) and patient are involved’ [67]. A CMR is effective in detecting and solving drug related problems (DRPs). Setting health related goals in consultation with the patient contributes to the achievement of positive clinic outcomes [67].

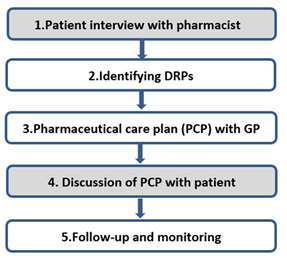

## 6. Final Remarks

To round off we share some general lessons we learned in the past years.

### 6.1. The Importance of Support by Management

The preferred educational methods of teaching and assessment (small group training and OSCEs) are time consuming and costly. It is important that the management of a school of pharmacy supports this, otherwise it is difficult to implement. Teachers at schools of pharmacy who are responsible for the development, implementation and provision of communication skills training have to consider how they can achieve maximum results given the opportunities (and barriers) in their own school.

### 6.2. Strive for Quality and Effectiveness

One should not compromise on the quality and effectiveness of the chosen teaching and assessment methods. When the activities are not effective it can be demotivating for staff and students, resulting in less support for this topic in the curriculum.

### 6.3. The Importance of Supportive and Qualified Staff

Support of the teaching staff is a prerequisite to integrate communication training in the pharmacotherapy courses (learning in context). The teachers should be qualified, preferably in both communication and pharmacotherapy. We experience difficulty in recruiting enough qualified and motivated pharmacists. There is also a kind of competition with other educational activities for which their competences are needed. Therefore, in our institution communication skills trainers and pharmacists/teachers work closely together as a second-best option. 

#### 6.3.1. Using the Experience and Expertise of Others

In the medical field there is a long history with teaching students about patient communication. Therefore, valuable expertise and experiences can be obtained from professional organizations such as the Association for Medical Education in Europe (AMEE), the International Association for Communication in Healthcare (EACH) and the American Academy for Communication in Healthcare (AACH) and their national counterparts.

#### 6.3.2. A Curriculum Is Never Finished

Our communication curriculum developed over the last 40 years and there is always room for improvement. For example, although we appreciate working with simulation patients, we think it would be a valuable experience if students also meet with real patients in an earlier stage of the curriculum (before the internships). We expect that meeting with real patients will motivate our students even more, because they experience the relevance.

To conclude, designing a curriculum on patient centered communication is a challenging but rewarding process. Rewarding because students are prepared for their future role as patient centered pharmacist. And because, according to our students, learning and assessment of communication is sometimes stressful but it is also instructive, realistic and fun.

## Figures and Tables

**Table 1:** Description of the stages of Miller’s Pyramid related to learning objectives and methods for patient centered communication (italic: activities not used in our curriculum).

Stages	Learning Objective	Teaching/Learning Method	Assessment Method (Formative and Summative)
1. Knowing what and why	Gaining insight in: underlying communication models, principles or methods the background and the goals of specific consultations	Self-study: - literature, e-learning modules *Lectures or groupwork for reflection and discussion*	Written reflection assignment *Written exam*
2. Knowing how	Understanding: how to perform a specific skill or method the goal and structure of specific consultations	Demonstration by experts (life or video): - observing and reflecting on examples of the skillSelf-study: - literature, e-learning modules, serious game/online simulation Small group training: - practicing specific skills or methods - roleplay of (parts of) consultations to discover difficulties and solutions to deal with the difficulties	Written reflection assignment *Written exam*
3. Showing	Demonstrating: the skill or method according to the standards making use of the acquired knowledge often in a safe and regulated setting	Small group training: - roleplay of (parts of) consultations to practice the desired behavior Formative assessment Video feedback and reflection assignments Simulation pharmacy	Objective Structured Clinical Exam (OSCE) Video-feedback Feedback on roleplay
4. Does	Performing: the skill or method in a qualified way in a personal authentic manner in real life settings and increasingly complex situations	Internships: - practicing patient centered communication and receiving feedback from their supervising pharmacists Portfolio: - collect data on own professional and personal development - reflect on own competences	Internship: assessment of learning activities regarding patient centered communication Assessment of student’s portfolio

**Table 2:** Overview of topics regarding pharmaceutical consultations on prescription medicines.

Topic *	Learning Objectives the Student …	Teaching/Learning Activities
1. Standard first prescription consultation	applies the basic structure of a first prescription consultation; provides essential information based on the characteristics of the medicine; gives information and advice in a clear, unambiguous and dosed manner and uses teach back; reflects on ways to discuss precarious subjects professionally with patients.	Two lectures on pharmaceutical care and pharmaceutical consultations Two preparatory e-modules Small group training (12–15 students); practicing with other students led by teacher/practicing pharmacist Workshop (12–15) on applying pharmaceutical knowledge during consultations Formative OSCE Written reflection assignment on the video recordings of the OSCE Practicing with a simulation patient (individually)
2. Standard second prescription consultation	applies the basic structure of second prescription consultation; uses techniques of active listening, (especially open questioning) to gather relevant information about patient’s situation and needs; analyses simple pharmaceutical problems/concerns of patients in order to give appropriate information and advice.	One preparatory e-module Small group training (12–15); practicing with other students led by teacher/practicing pharmacist Formative OSCE Written reflection assignment on the video recordings of the OSCE Practicing with a simulation patient (individually)
3. Involving a distracted patient in the consultation, (e.g., absentminded, in a hurry or in pain)	applies the basic structure of a first prescription consultation; involves distracted patients by making contact, to ensure they are willing to take part in the conversation; adapts their communication style to the patient’s to build rapport.	One preparatory e-module Small group training (6–8); practicing with a simulation patient led by communication skills trainer Formative OSCE Written reflection assignment on the video recordings of the OSCE Practicing with a simulation patient (individually)
4. Coping with a talkative patient	applies the basic structure of a second prescription consultation; takes charge of a patient who is talkative in a polite and respectful way; adapts their communication style to the patient’s to build rapport.	One preparatory e-module Small group training (6–8); practicing with a simulation patient led by communication skills trainer Formative OSCE Written reflection assignment on the video recordings of the OSCE Practicing with a simulation patient (individually)
5. Handling patient’s emotion	adequately deals with a patient’s emotions about illness or medication; breaks bad news in a professional manner; is listening invitingly and attentively, allowing the patient to share their concerns and ideas.	Two lectures on health psychology and patient centered communication One preparatory e-module Small group training (6–8); practicing with a simulation patient led by communication skills trainer Formative OSCE Practicing with a simulation patient (individually)
6. Addressing (potential) non-compliance	confronts the patient in a respectful way with the potential non-compliance; deals effectively with patient’s resistance, tries to avoid raising patient’s resistance; explores and analyses the patient’s (biopsychosocial) concern/problem together with the patient; decides with the patient on the best solution in their situation; respects the patient’s autonomy and personal responsibility.	One preparatory e-module Small group training (6–8); practicing with a simulation patient led by communication skills trainer Formative OSCE Practicing with a simulation patient (individually)

* These topics are also the topics of the formative and summative OSCE-stations (OSCE: Objective Structured Clinical Exam).

**Table 3:** Model for pharmaceutical consultations (according to the Dutch guideline ‘pharmaceutical consultations’).

Phase	Content	Continuously throughout the Consultation
Initiating the consultation	Establishing the relationship Identifying the reasons for the consultation Agenda setting	Building the relationship
Gathering information	Exploring patient’s needs and perspective Reaching a shared understanding of the problem	
Explanation, advice and decision making	Providing relevant and clear information and advice Shared decision making on management plan	Providing structure
Closing the consultation	Enabling self-management Agreement check Follow up	

**Table 4:** Overview of learning objectives and educational activities on pharmaceutical consultations on self-care medication.

Learning Objectives the Student …	Teaching/Learning Activities
analyses the patient’s situation and needs adequately using the WHAM questions *; provides concrete and appropriate information and advice based on professional guidelines (medical advice, lifestyle advice, referral to the doctor and/or additional information); determines in which situations exceptions should be made to the general guidelines; gives information and advice in a clear, unambiguous and dosed manner and uses teach back; decides with the patient on the best solution in their situation; respects the patient′s autonomy and personal responsibility; recognizes the limits and responsibilities of their profession and refers to the doctor if necessary.	Introductory lecture on self-care medication, Dutch regulations and examples of patient cases Self-study of six professional Dutch guidelines on selfcare for allergic rhinitis, diarrhea, emergency contraception, athlete’s foot, upset stomach, pain medication: NSAIDs including online tests on the guidelines (if available) Small group training (12–15 students) with focus on both communication skills and pharmacotherapy; practicing with other students led by teacher/practicing pharmacist Practicing with simulation patients in the simulation pharmacies Practicing with a simulation patient (individually; 0.5 h) (formative assessment) Practicing real-life consultations during their internship

* WHAM questions: Who is it for?; How long have the symptoms been present, (what are the symptoms)?; What Actions have been taken so far?; What other Medication is being used at present?

**Table 5:** Overview of the educational activities concerning clinical medication review (CMR).

Learning Goals the Student…	Teaching/Learning Activities
**CMR**	
has insight in the whole process of CMR, the necessary competences and the role of the pharmacist prepares a questionnaire (concerning usage, effects and side effects) is aware of the importance of effective questioning in order to elicit all relevant information is able to identify and analyze drug related problems (DRP)	Introductory lecture on the medication review process Group work introduction on CMR - preparing a questionnaire, which is practiced using two paper patient cases - interview in couples or trios with a simulation patient on their drug related problems - reporting and discussing the outcomes of the interviews plenary Five sessions of group work on CMR (discussing paper patient cases); this is organized parallel with the activities on the patient interview
**Patient Interview**	
builds a therapeutic relationship with the patient deals adequately with the patient’s emotions and attitudes obtains relevant information through open interviewing identifies the patient’s drug related problems elicits and identifies relevant information relates the obtained information with their own pharmaceutical knowledge deals adequately with a patient’s emotions and attitudes structures (e.g., summarizing, sign posting) the patient interview knows to what extent they can independently conduct a (simple) patient interview gains insight into personal strengths and weaknesses	Introductory lecture on the structure of the patient interview and necessary competences (recently replaced by an e-module) Small group training (6–8) on the introduction and intake; practicing with a simulation patient led by communication skills trainer Small group training (6–8) on the medication overview, anamnesis and closure; practicing with a simulation patient led by a pharmacist (teacher) Training session: taking a full patient interview - carrousel: in turn three students interview a different simulation patient - giving and receiving peer feedback and feedback from the simulation patient
practices real-life situations in preparation for their future job as a pharmacist	Internship (community pharmacy) year 1 - one patient interview is assessed by the internship supervisor - students can practice more often
feels capable to conduct a patient interview independently gains insight into personal strengths and weaknesses	Training session: taking a full patient interview - optional rehearsal for community pharmacy internship year 3 - 20–25% of the students participate
***Discussing the pharmaceutical care plan with the patient***	
has insight into the basic principles of motivational interviewing applies the core skills of MI has experienced the effect of MI on behavioral change is aware how communication skills of MI contribute to fulfilling the role of a coach	E-module and training session on motivational interviewing (MI) Applied for discussing the treatment plan with the patient Small group training (6–8); practicing with a simulation patient led by a communication skills trainer
practices real-life situations in preparation for their future job as a pharmacist	Internship (community pharmacy) year 3 - students observe at least one CMR and perform one CMR under supervision, including the patient interview and discussing the pharmaceutical care plan with the patient

**Table 6:** Conversation model for the patient interview (step 1).

Phase	Content
Introduction	Acquaintance Introduction on the aim, agenda and follow-up
Intake *	Open interview on patient’s personal situation, their experience with diseases and medication, preferences, questions and problems
Medication overview *	Patient explains how they use their medication on an average day (giving insight in self-management, practical problems and medication adherence)
Anamnesis *	Structured interview on specific topics to get a full understanding of the biomedical situation of the patient (medical condition, usage, effect and side effects of medication)
Closure	Summarizing the identified problems and questions Explaining follow-up

* In these phases drug related problems can be identified.

## Data Availability

Data sharing not applicable.

## References

[1] Droege M. (2003). The role of reflective practice in pharmacy. Educ. Health Chang. Learn. Pract..

[2] Hepler C.D., Strand L.M. (1990). Opportunities and responsibilities in pharmaceutical care. Am. J. Hosp. Pharm..

[3] International Pharmaceutical Federation (2019). Strategic Plan 2019 to 2024.

[4] De Oliveira D.R., Shoemaker S.J. (2006). Achieving patient centeredness in pharmacy practice: Openness and the pharmacist’s natural attitude. J. Am. Pharm. Assoc..

[5] Sánchez A.M. (2011). Teaching patient-centered care to pharmacy students. Int. J. Clin. Pharm..

[6] Ilardo M.L., Speciale A. (2020). The community pharmacist: Perceived barriers and patient-centered care communication. Int. J. Environ. Res. Public Heal..

[7] Illingworth R. (2010). What does ‘patient-centred’ mean in relation to the consultation?. Clin. Teach..

[8] Koster A.S., Mantel-Teeuwisse A., Woerdenbag H.J., Mulder W., Wilffert B., Schalekamp T., Buurma H., Wilting I., Westein M. (2020). Alignment of CanMEDS-based undergraduate and postgraduate pharmacy curricula in The Netherlands. Pharmacy.

[9] Wolfe A. (2001). Institute of Medicine Report: Crossing the quality chasm: A new health care system for the 21st century. Policypoliti-Nurs. Pract..

[10] Mead N., Bower P. (2002). Patient-centred consultations and outcomes in primary care: A review of the literature. Patient Educ. Couns..

[11] Stewart M., Brown J., Weston W., Mcwhinney I., McWilliam C., Freeman T. (2003). Patient-Centered Medicine: Transforming the Clinical Method.

[12] Wolters M., Van Hulten R., Blom L., Bouvy M.L. (2017). Exploring the concept of patient centred communication for the pharmacy practice. Int. J. Clin. Pharm..

[13] Hyvärinen M.-L., Tanskanen P., Katajavuori N., Isotalus P. (2010). A method for teaching communication in pharmacy in authentic work situations. Commun. Educ..

[14] Makoul G. (2001). Essential elements of communication in medical encounters: The Kalamazoo consensus statement. Acad. Med..

[15] Hargie O., Morrow N.C., Woodman C. (2000). Pharmacists’ evaluation of key communication skills in practice. Patient Educ. Couns..

[16] Brown R.F., Bylund C.L. (2008). Communication skills training: Describing a new conceptual model. Acad. Med..

[17] Cegala D.J., Broz S.L. (2002). Physician communication skills training: A review of theoretical backgrounds, objectives and skills. Med Educ..

[18] Kurtz S.M., Silverman J.D. (1996). The Calgary-Cambridge Referenced Observation Guides: An aid to defining the curriculum and organizing the teaching in communication training programmes. Med. Educ..

[19] Blind C.S., Daemen B.J.G., Faber A. Mulder-Wildemors LGM, Meijvis VAM. KNMP-Richtlijn Consultvoering.

[20] Koster A.S., Schalekamp T., Meijerman I. (2017). Implementation of Competency-Based Pharmacy Education (CBPE). Pharmacy.

[21] Biggs J. (1996). Enhancing teaching through constructive alignment. High. Educ..

[22] Bachmann C., Abramovitch H., Barbu C.G., Cavaco A.M., Elorza R.D., Haak R., Loureiro E., Ratajska A., Silverman J., Winterburn S. (2013). A European consensus on learning objectives for a core communication curriculum in health care professions. Patient Educ. Couns..

[23] Deci E.L., Ryan R.M. (2000). The “what” and “why” of goal pursuits: Human needs and the self-determination of behavior. Psychol. Inq..

[24] Robinson J.D., Persky A.M. (2020). Developing self-directed learners. Am. J. Pharm. Educ..

[25] Beardsley R.S. (2001). Communication skills development in colleges of pharmacy. Am. J. Pharm. Educ..

[26] Svensberg K., Björnsdottir I., Wallman A., Sporrong S.K. (2017). Nordic pharmacy schools’ experience in communication skills training. Am. J. Pharm. Educ..

[27] Wallman A., Vaudan C., Sporrong S.K. (2013). Communications training in pharmacy education, 1995–2010. Am. J. Pharm. Educ..

[28] Emmanuelle S., Chung E., Sakharkar P., Law A.V. (2013). Instruction and assessment of student communication skills in US and Canadian pharmacy curricula. Curr. Pharm. Teach. Learn..

[29] Miller G.E. (1990). The assessment of clinical skills/competence/performance. Acad. Med..

[30] Cruess R.L., Cruess S.R., Steinert Y. (2016). Amending Miller’s pyramid to include professional identity formation. Acad. Med..

[31] Bell C., Paterson J., Murison P., Warman S.M. (2014). How do we learn?. InPractice.

[32] Berkhof M., van Rijssen H.J., Schellart A.J., Anema J.R., van der Beek Allard J. (2011). Effective training strategies for teaching communication skills to physicians: An overview of systematic reviews. Patient Educ. Couns..

[33] Van Dalen J., Bartholomeus P., Kerkhofs E., Lulofs R., Van Thiel J., Rethans J.J., Scherpbier A.J., Van Der Vleuten C.P. (2001). Teaching and assessing communication skills in Maastricht: The first twenty years. Med. Teach..

[34] Taylor P.J., Russ-Eft D.F., Chan D.W.L. (2005). A meta-analytic review of behavior modeling training. J. Appl. Psychol..

[35] Latham G.P., Saari L.M. (1979). Application of social-learning theory to training supervisors through behavioral modeling. J. Appl. Psychol..

[36] Schönrock-Adema J. (2002). De Ontwikkeling En Evaluatie Van Een Zelfinstructieprogramma Voor Een Training In Basisgespreksvaar-Digheden. Ph.D. Thesis.

[37] Jin H.K., Choi J.H., Kang J.E., Rhie S.J. (2018). The effect of communication skills training on patient-pharmacist communication in pharmacy education: A meta-analysis. Adv. Health Sci. Educ..

[38] Jin H.K., Park S.H., Kang J.E., Choi K.S., Kim H.A., Jeon M.S., Rhie S.J. (2019). The influence of a patient counseling training session on pharmacy students’ self-perceived communication skills, confidence levels, and attitudes about communication skills training. BMC Med. Educ..

[39] Kubota R., Shibuya K., Tanaka Y., Aoki M., Shiomi M., Ando W., Otori K., Komiyama T. (2018). Clinical pharmacy education in Japan: Using simulated patients in laboratory-based communication-skills training before clinical practice. Pharmacy.

[40] Rao D. (2011). Skills development using role-play in a first-year pharmacy practice course. Am. J. Pharm. Educ..

[41] Blom L., Wolters M., Hoor-Suykerbuyk M.T., Van Paassen J., Van Oyen A. (2011). Pharmaceutical education in patient counseling: 20h spread over 6 years?. Patient Educ. Couns..

[42] Gartmeier M., Bauer J., Fischer M.R., Hoppe-Seyler T., Karsten G., Kiessling C., Möller G.E., Wiesbeck A., Prenzel M. (2015). Fostering professional communication skills of future physicians and teachers: Effects of e-learning with video cases and role-play. Instr. Sci..

[43] Hess R., Hagemeier N.E., Blackwelder R., Rose D., Ansari N., Branham T. (2016). Teaching communication skills to medical and pharmacy students through a blended learning course. Am. J. Pharm. Educ..

[44] Wolters M., Van Paassen J.G. Pharmacy students appreciate e-learning modules to prepare for communication skills training. Proceedings of the International Conference on Communication in Healthcare, ICCH.

[45] Rickles N.M., Tieu P., Myers L., Galal S., Chung V. (2009). The impact of a standardized patient program on student learning of com-munication skills. Am. J. Pharm. Educ..

[46] May W., Park J.H., Lee J.P. (2009). A ten-year review of the literature on the use of standardized patients in teaching and learning: 1996–2005. Med. Teach..

[47] Schlegel C., Woermann U., Shaha M., Rethans J.J., van der Vleuten C. (2012). Effects of communication training on real practice per-formance: A role-play module versus a standardized patient module. J. Nurs. Educ..

[48] Lin E.C., Chen S., Chao S., Chen Y. (2013). Using standardized patient with immediate feedback and group discussion to teach inter-personal and communication skills to advanced practice nursing students. Nurse Educ. Today.

[49] Bosse H.M., Schultz J.-H., Nickel M., Lutz T., Möltner A., Jünger J., Huwendiek S., Nikendei C. (2012). The effect of using standardized patients or peer role play on ratings of undergraduate communication training: A randomized controlled trial. Patient Educ. Couns..

[50] Gillette C., Stanton R.B., Anderson H.G. (2017). Student performance on a knowledge-based exam may predict student ability to communicate effectively with a standardized patient during an objective structured clinical examination. Curr. Pharm. Teach. Learn..

[51] Newble D. (2004). Techniques for measuring clinical competence: Objective structured clinical examinations. Med. Educ..

[52] Khan K.Z., Ramachandran S., Gaunt K., Pushkar P. (2013). The objective structured clinical examination (OSCE): AMEE Guide no. 81. Part I: An historical and theoretical perspective. Med. Teach..

[53] Pell G., Fuller R., Homer M., Roberts T. (2010). How to measure the quality of the OSCE: A review of metrics–AMEE Guide no. 49. Med. Teach..

[54] Sturpe D. (2010). Objective structured clinical examinations in doctor of Pharmacy programs in the United States. Am. J. Pharm. Educ..

[55] Fens T., Dantuma-Wering C.M., Taxis K. (2020). The pharmacy game-GIMMICS^®^ a simulation game for competency-based education. Pharmacy.

[56] Watson M.C., Bond C.M., Grimshaw J.M., Johnston M. (2006). Factors predicting the guideline compliant supply (or non-supply) of non-prescription medicines in the community pharmacy setting. BMJ Qual. Saf..

[57] Sabate E. (2003). Adherence to Long Term Therapies. Evidence to Action.

[58] Jokanovic N., Tan E.C., van den Bosch D., Kirkpatrick C.M., Dooley M.J., Bell J.S. (2016). Clinical medication review in Australia: A systematic review. Res. Soc Adm. Pharm..

[59] Bulajeva A., Labberton L., Leikola S., Pohjanoksa-Mäntylä M., Geurts M., De Gier J., Airaksinen M. (2014). Medication review practices in European countries. Res. Soc. Adm. Pharm..

[60] Hatah E., Braund R., Tordoff J., Duffull S.B. (2014). A systematic review and meta-analysis of pharmacist-led fee-for-services medication review. Br. J. Clin. Pharmacol..

[61] Bryant L.J., Coster G., Gamble G.D., McCormick R.N. (2011). The General Practitioner–Pharmacist Collaboration (GPPC) study: A ran-domised controlled trial of clinical medication reviews in community pharmacy. Int. J. Pharm. Pract..

[62] Burns A. (2008). Medication therapy management in pharmacy practice: Core elements of an MTM service model (version 2.0). J. Am. Pharm. Assoc..

[63] Blenkinsopp A., Bond C., Raynor D.K. (2012). Medication reviews. Br. J. Clin. Pharmacol..

[64] NHG (2012). NHG, en Verzorging, Landelijk Expertisecentrum Verpleging. Multidisciplinaire Richtlijn Polyfarmacie Bij Ouderen 2012.

[65] Meijer W.M., Daemen B. (2013). KNMP-Richtlijn Medicatiebeoordeling.

[66] Miller W.R., Rollnick S. (2009). Ten things that motivational interviewing is not. Behav. Cogn. Psychother..

[67] Lonie J.M., Austin Z., Nguyen R., Gill I., Lucas C. (2017). Pharmacist-based health coaching: A new model of pharmacist-patient care. Res. Soc. Adm. Pharm..

[68] Sisson E., Kuhn C. (2009). Pharmacist roles in the management of patients with type 2 diabetes. J. Am. Pharm. Assoc..

